# Distribution and efficacy of dolutegravir-based regimens in the main HIV outpatient care in Caracas, Venezuela

**DOI:** 10.1186/s12981-025-00811-y

**Published:** 2025-10-27

**Authors:** María F. Alvarado-Bruzual, Víctor A. Mendoza-Merlo, Jesus A. Martinez-Torres, Luis J. Guerra-Reyes, Rafael N. Guevara, Martín Carballo, María Carolyn Redondo, María E. Landaeta, David A. Forero-Peña

**Affiliations:** 1 Department of Infectious Diseases, Hospital Universitario de Caracas, Caracas, Venezuela; 2 Biomedical Research and Therapeutic Vaccines Institute, Caracas, Venezuela; 3 “Luis Razetti” School of Medicine, Universidad Central de Venezuela, Caracas, Venezuela; 4 Infectious Diseases Research Department, Infectus, Bogota, Colombia

**Keywords:** HIV, Antiretroviral therapy, Venezuela, Dolutegravir, Tenofovir, PLHIV

## Abstract

Venezuela experienced an interruption in antiretroviral therapy (ART) from 2016 to 2018. Although in early 2019, the dolutegravir (DTG) based regimens were implemented for HIV treatment in Venezuela, few studies have evaluated the efficacy of these regimens. This cross-sectional study describes the utilization, switches and efficacy of ART regimens in the main HIV outpatient care in Venezuela in 2024. Data from 1,998 patient records revealed that the dolutegravir/lamivudine/tenofovir (DTG/3TC/TDF) regimen was predominantly used (85.5%). A high viral suppression rate of over 90% was documented for all DTG-based regimens, with no significant difference found between the main regimen (DTG/3TC/TDF) and its alternatives: DTG/emtricitabine/tenofovir alafenamide (TAF), abacavir/3TC + DTG and, DTG/3TC. Overall, almost all switches (97.1%) were made toward DTG/FTC/TAF. Osteoporosis was the main reason for switching treatments (80.1%).

## Background

By the end of 2023, approximately 2.3 million people across the Latin American region were living with the human immunodeficiency virus (HIV). During the same period, 120,000 new infections occurred, resulting in 30,000 HIV-related deaths [1]. Despite global progress in reducing HIV mortality by 52% since 2010, Latin America experienced a more modest reduction of only 28%. According to World Health Organization data for 2023, approximately 100,000 people live with HIV (PLHIV) in Venezuela, with 7,600 new cases and 1,500 deaths reported by 2022 [2].

Since 2016, Venezuela has experienced the highest rate of antiretroviral therapy (ART) interruptions among Latin American countries, peaking between 2017 and 2018, when only 16% of patients were receiving treatment in April 2018 [3]. This led to a mass exodus of Venezuelan migrants with HIV, straining the health systems of receiving countries [4–6]. In 2019, nationwide ART access was re-established primarily through a dolutegravir (DTG)-based regimen, funded by the “Master Plan for Strengthening the Response to HIV, Tuberculosis, and Malaria,” with ongoing funding by the World Bank [7]. The efficacy of DTG-based regimens is estimated to be 93–99% at 48 weeks [8, 9] and a lower risk of discontinuation due to adverse effects [10] when compared to previously prevalent ART regimens (e.g., efavirenz, atazanavir/ritonavir, darunavir/ritonavir, and raltegravir).

Among the challenges, the impact of the health crisis on the HIV continuum of care in Venezuela remains underexplored [11]. Despite the reported effectiveness and safety of DTG-based regimens, in Venezuela few studies —with limited PLHIV samples— have analyzed their efficacy [12, 13], especially in a context of empirical switches due to previous shortages [3]. Furthermore, the use of alternative regimens in case of comorbidities such as renal disease or osteoporosis has not been explored (e.g., regimens with tenofovir alafenamide, abacavir, or efavirenz). Based on experience in the main outpatient care center for PLHIV in Venezuela, this cross-sectional study aims to describe the use of different ART regimens and attempt to estimate their efficacy, as well as to identify the frequency of ART regimen switches and their main reasons.

## Methods

### Study design and patients

**Fig. 1:**
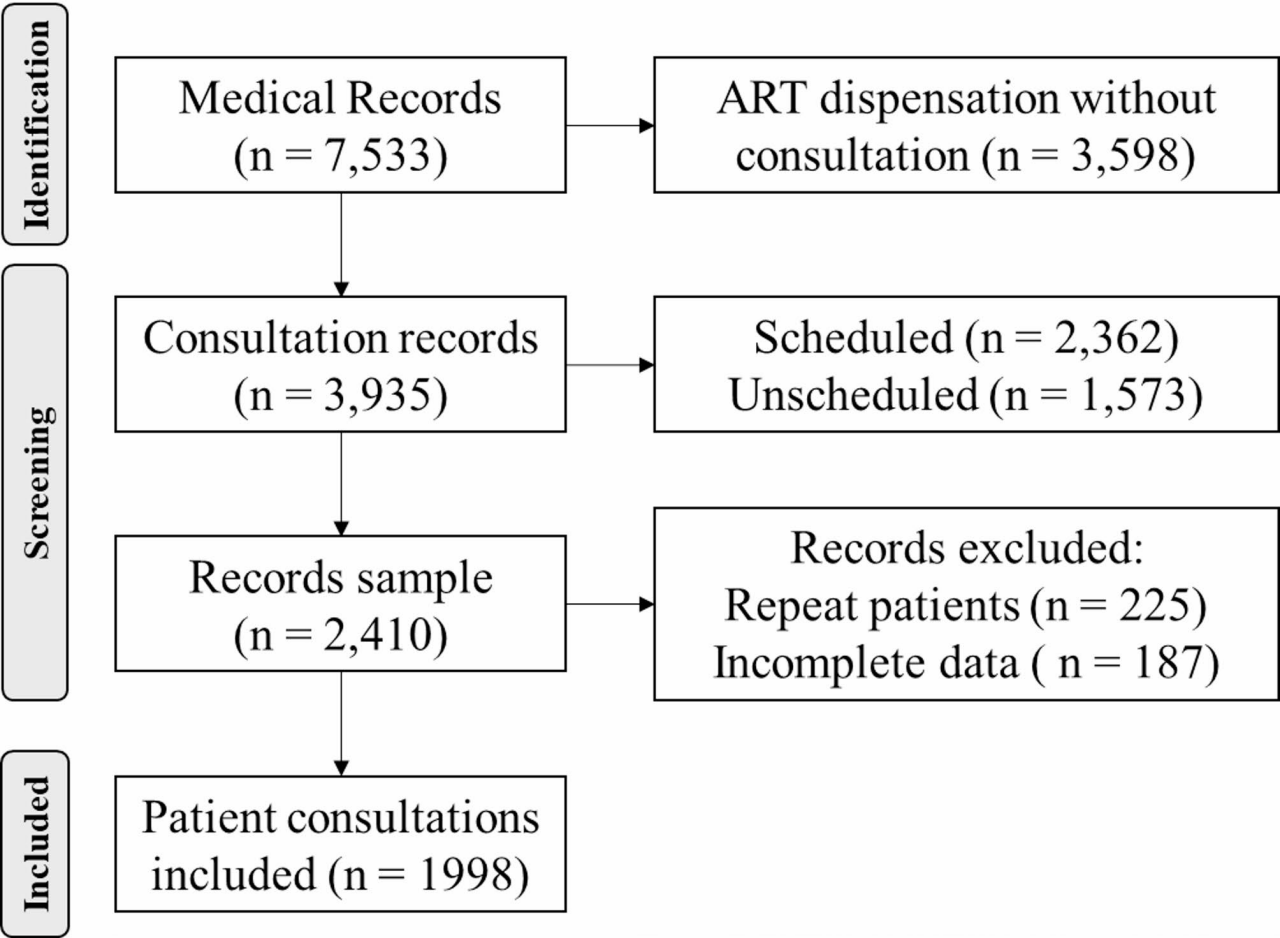
Flowchart of the participants’ selection

A cross-sectional study was conducted between January and December 2024 at the outpatient clinic of the Infectious Diseases Department of the University Hospital of Caracas, Venezuela. This specialized outpatient clinic has been providing care to patients with HIV infection since 1990 and is considered the largest in the country, having seen a total of 6,350 patients in 2022. The study included consecutive medical visits of patients aged 13 years or older with known or newly diagnosed HIV infection without missing data on prescribed ART (Fig. 1). To analyze this population with a 99% confidence interval and a margin of error of 5%, a sample size of at least 604 participants was required. A non-probabilistic convenience sampling method was used.

### Survey design and data collection

A data collection form was designed to collect sociodemographic and treatment regimen data. Information on the current ART regimen at the time of the evaluated consultation were recorded, as well as changes in ART regimens prescribed by the physician and the reasons for such switches. The last available viral loads and CD4+ cell counts were recorded. From the total registry obtained, we excluded inconsistent or incomplete records and repeat patients, in such cases, we selected the record with the most information, such as details of viral load.

### Data analysis

Participant data were summarized using descriptive statistics, including median, interquartile range (IQR), and/or frequency, percentage (%). The normality of numeric variables was assessed using the Kolmogorov-Smirnov test. Univariable analyses were performed using the Mann-Whitney U test for numerical variables (given their non-normal distribution), and Pearson’s chi-squared and Fisher’s exact tests for categorical variables. *P* values less than 0.05 were considered significant. Statistical analyses were performed using the Statistical Package for the Social Sciences version 26 (International Business Machines Corporation, Armonk, NY, USA).

## Results

A total of 2,410 patient records were initially reviewed for the study. After a screening, 412 records were excluded due to incomplete data (*n* = 187) or repeat patients (*n* = 225). The patient records selection process is detailed in the flowchart (Fig. 1). A total of 1,998 patients were included in the study. The median age was 44 years (IQR 33–55), ranging from 13 to 88 years, and most were male (70.8%). At the time of the evaluated consultation, all patients were receiving a dolutegravir-based regimen. The most common regimen was DTG/3TC/TDF (85.5%) (Acriptega^®^), which is the standard first-line therapy in the country unless contraindicated (e.g., in cases of renal disease or high risk of bone fracture) [14]. Other regimens included: DTG/emtricitabine (FTC)/tenofovir alafenamide (TAF) (9.1%) (Kocitaf^®^), abacavir (ABC)/3TC + DTG (3.3%), and DTG/3TC (2.1%) (Table 1).

**Fig. 2:**
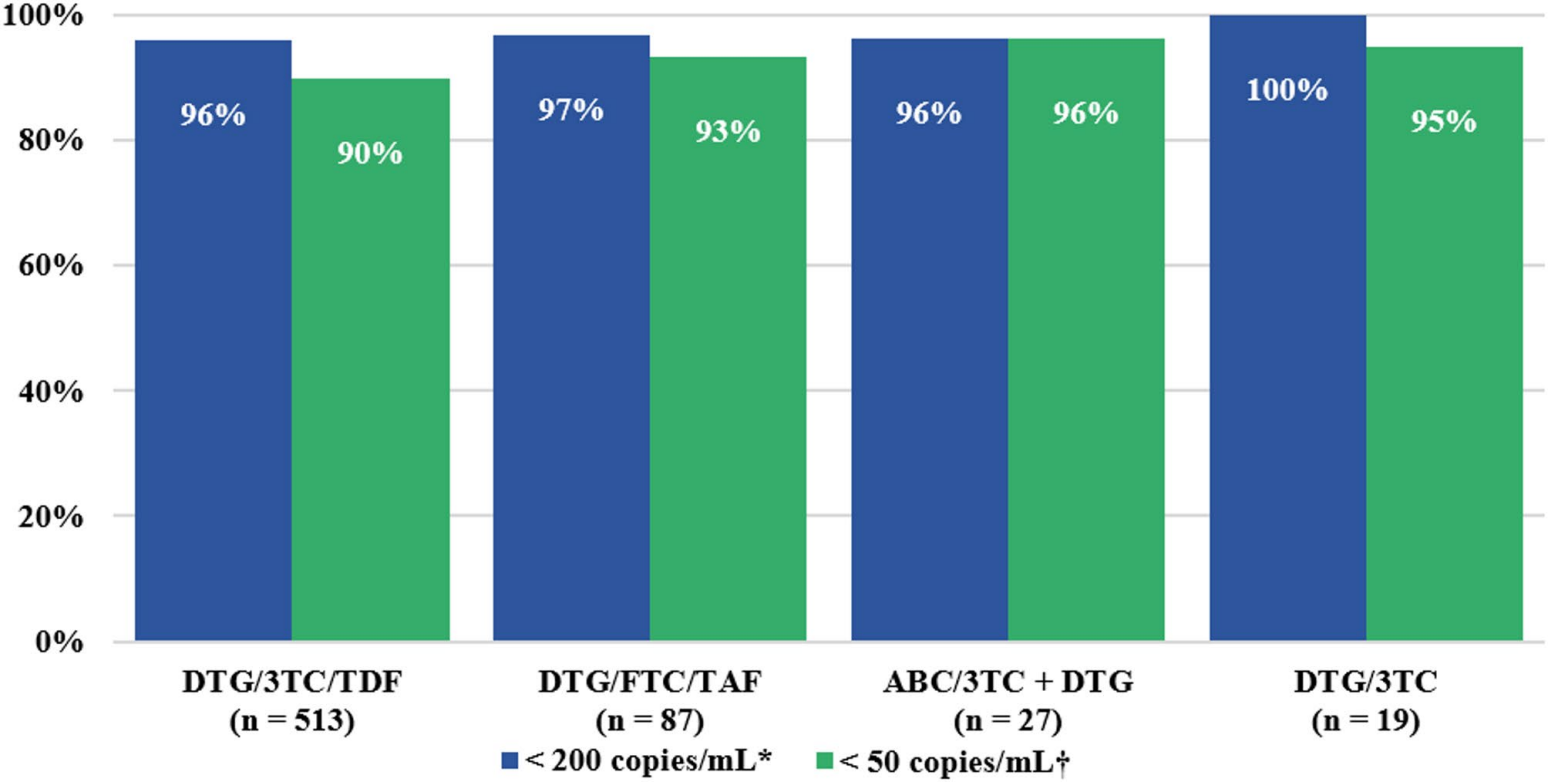
Efficacy of dolutegravir-based regimens in treatment-experienced HIV patients at the University Hospital of Caracas, 2024. Differences in viral suppression rates were compared across the four regimen groups. No significant differences were observed between regimens for viral loads < 200 copies/mL (^*^p = 1 by Fisher’s exact test) or < 50 copies/mL (^†^*p* = 0.464 by Pearson’s chi-square test). *3TC*: lamivudine; *ABC*: abacavir; *DTG*: dolutegravir; *FTC*: emtricitabine; *TAF*: tenofovir alafenamide; *TDF*: tenofovir disoproxil fumarate

The frequency of women was higher in the alternative ART group compared to the DTG/3TC/TDF regimen (42.8% vs. 26.9%; *p* < 0.001). A significant association was found between DTG/3TC/TDF therapy and being aged 20–34 years (*p* < 0.001) as well as 35–50 years (*p* < 0.001), while alternative ART was significantly associated with the > 50 age group (*p* < 0.001). No significant differences were found between those with and without viral suppression, nor in CD4 count (Table 1). Viral load was only available in 654 patients (32.7%), documenting a viral suppression rate of over 90% in all regimens (Fig. 2).

During the study period, 116 switches in antiretroviral regimens were recorded, mainly by switching them to DTG/FTC/TAF (97.5%), the reasons for the switches being for osteoporosis (*n* = 93; 80.1%), kidney impairment (*n* = 22; 19.0%) and resistance to antiretroviral therapy (*n* = 1; 0.9%). A total of 19 pregnancies were recorded, and these were managed with DTG/3TC/TDF.

**Table 1:** Characteristic and antiretroviral treatment of PLHIV selected at the university hospital of Caracas in 2024

Characteristic *n* (%)		Total *n* = 1998 (100)	DTG/3TC/TDF *n* = 1708 (85.5)	Alternative ART^#^ *n* = 290 (14.5)	*P* value
**Sex**					< 0.001*
	Female	584 (29.2)	460 (26.9)	124 (42.8)	
	Male	1414 (70.8)	1248 (73.1)	166 (57.2)	
**Age**, years, median (IQR)		44 (33–55)	41 (32–51)	59 (53–65)	**< 0.001** ^†^
**Age groups**, years					< 0.001* ^‡^
	< 20	24 (1.2)	23 (1.4)	1 (0.3)	
	20–34	538 (27.1)	529 (31.2)^‡^	9 (3.1)	
	35–50	744 (37.4)	700 (41.2)^‡^	44 (15.2)	
	> 50	682 (34.3)	446 (26.3)	236 (81.4)^‡^	
**ART experience**					< 0.001*
	Naïve	367 (18.4)	362 (21.2)	5 (1.7)	
	No Naïve	1631 (81.6)	1346 (78.8)	285 (98.3)	
**No naïve viral load**, copies/mL		646 (32.3)			0.415*
	≤ 50	585 (90.6)	460 (89.7)	125 (89.7)	
	50–199	36 (5.6)	32 (6.2)	4 (3)	
	200–999	9 (1.4)	7 (1.4)	2 (1.5)	
	≥ 1000	16 (2.5)	14 (2.7)	2 (1.5)	
**No naïve CD4 count**, cells/µL		188 (9.4)			0.772*
	< 200	10 (5.3)	8 (5.1)	2 (6.3)	
	200–499	56 (29.8)	45 (28.8)	11 (34.4)	
	≥ 500	122 (64.9)	103 (66)	19 (59.4)	

*Pearson’s chi-square test; ^†^Mann–Whitney U test; ^‡^Significant association only between DTG/3TC/TDF and 20–34 years (*p* < 0.0001), DTG/3TC/TDF and 35–50 years (*p* < 0.0001), and between Alternative ART and > 50 years (*p* < 0.0001) with an *α* = 0.00625 by Bonferroni correction

^#^Alternative ART includes the following regimens: DTG/FTC/TAF, ABC/3TC + DTG, and DTG/3TC. *3TC*: lamivudine; *ABC*: abacavir; *ART*: antiretroviral treatment; *DTG*: dolutegravir; *FTC*: emtricitabine; *IQR*: interquartile range; *TAF*: tenofovir alafenamide; *TDF*: tenofovir disoproxil fumarate

## Discussion

Our results show the distribution of the main antiretroviral regimens used by a large number of PLHIV treated at the country’s referral center, and report efficacy of over 90% for all dolutegravir-based regimens. The use of DTG has been studied in treatment-naïve and treatment-experienced HIV patients, and the results are mostly very favourable, placing it relatively ahead of other antiretrovirals. In FLAMINGO [15] and SPRING-2 [16] studies, 90% and 88% of naïve patients achieved < 50 copies/mL at week 48, respectively. The DAWNING [17] and NADIA [18] studies tested dolutegravir-based regimens in previously pre-treated patients, including those with virologic failure, achieving viral suppression of 84% and 90.2%, respectively. In all these trials, dolutegravir-based regimens were found to be superior to other ART regimens.

After a pronounced shortage of antiretroviral treatment in Venezuela during 2018, DTG/3TC/TDF became the first-line treatment [14]. Thanks to the effort of Pan American Health Organization, The Global Fund to Fight AIDS, Tuberculosis and Malaria, UNAIDS, UNICEF and civil society, in 2019 Venezuela began to receive ART, mostly dolutegravir-based regimens; this meant that by the end of 2020, 91% of PLHIV were receiving DTG/3TC/TDF in fixed doses [19]. However, studies on these dolutegravir-based regimens in the country are limited. This is especially important in the context of PLHIV in Venezuela who restarted treatment empirically (without testing viral load due to unavailability) after interrupting medication due to stock shortages in 2018. Previously, in a small study with PLHIV in Caracas, we estimated an efficacy of DTG/3TC/TDF of 95.8% [13]. Another study, in Táchira, documented that viral suppression of DTG/3TC/TDF was 95.6% [12].

In this study we document that the most frequent alternative dolutegravir-based regimen was DTG/FTC/TAF and that its main reason for indication was osteoporosis, which is consistent with the National Institutes of Health Guidelines for the Use of Antiretroviral Agents [20] because TAF has less bone and kidney toxicity than TDF [21]. The documented efficacy of this TAF-containing regimen was over 93%, similar to that previously reported [22]. We report low use of DTG + 3TC, a recommended initial antiretroviral regimen for most people with HIV, with exceptions for people with HIV-1 RNA >500,000 copies/mL, coinfection with hepatitis B virus (HBV), or those who start treatment before the results of HIV reverse transcriptase genotypic resistance testing or HBV testing are available; this is likely related to the costs of these tests and the widespread use of DTG/3TC/TDF supported by The Global Fund in the country [19].

This study has several limitations: (1) The very low number of patients with available viral load (32.7%) and CD4 cell count data (9.4%) severely limited the analysis of these variables. (2) This study does not analyze data from patients who only collect prescriptions without attending consultations, who represent almost a third of outpatient care. (3) Although our sample is large and outpatient care is the largest in the country, our results only represent the central region of Venezuela. Also, it was not possible to analyze pharmacy data (drug dispensary). (4) The duration of antiretroviral treatment was not considered among the variables for analysis. Finally, (5) resistance testing for patients with detectable viral load was not available in this study.

Based on our findings and the limitations, several avenues for future research are suggested: prospective studies are needed to assess the long-term outcomes of the ART regimens used in our setting. Additionally, studies with the necessary logistical and financial support are required to ensure a higher number of viral load and CD4 cell count measurements, which would provide a more comprehensive analysis. Finally, it is crucial to investigate the reasons behind the high proportion of patients who do not attend medical consultations.

## Conclusions

In this large population of PLHIV analyzed in Venezuela, we found that all patients were on DTG-based regimens, with a clear predominance of DTG/3TC/TDF. The efficacy of these regimens was greater than 90%. The main alternative regimen was DTG/FTC/TAF due to its low bone and renal toxicity.

## Data Availability

The datasets supporting the conclusions of this study are not openly available due to sensitivity reasons, but are available from the corresponding author upon reasonable request.

## Abbreviations

3TC: Lamivudine
ABC: Abacavir
ART: Antiretroviral therapy
DTG: Dolutegravir
FTC: Emtricitabine
HBV: Hepatitis B virus
HIV: Human immunodeficiency virus
IQR: Interquartile range
PLHIV: People living with HIV
TAF: Tenofovir alafenamide
TDF: Tenofovir disoproxil fumarate
